# Considering mean and inequality health outcomes together: the population health performance index

**DOI:** 10.1186/s12939-018-0731-2

**Published:** 2018-02-17

**Authors:** David Kindig, Nicholas Lardinois, Yukiko Asada, John Mullahy

**Affiliations:** 1 University of Wisconsin-Madison Population Health Institute, 610 Walnut Street, 550 WARF, Madison, WI 53726 USA; 2 Dalhousie University Centre for Clinical Research, 5790 University Avenue, Room 405, Halifax, NS B3H 1V7 Canada

**Keywords:** Population health, Health outcomes, Index, Disparity, Inequality, Tradeoff

## Abstract

**Background:**

The purpose was to develop and test a population health measure that combines mean health outcomes and inequalities into a single GDP-like metric to help policymakers measure population health performance on both dimensions in one metric.

**Methods:**

The Population Health Performance Index is a weighted average of a mean index and an inequality index according to the user’s inequality aversion. We deploy this methodology for two combinations of health outcome and disparity domain: infant mortality by race and unhealthy days by education.

**Results:**

The PHPI is bounded between 0 and 1, and is comprised of a weighted average of two separate indices: a mean index and an inequality index, with 1 representing the ideal state of no ill health and no inequality and 0 representing the worst state in the U.S. PHPI values across states (neutral 50:50 weighting) vary between 0.60 (Massachusetts) to 0.17 (Delaware) for infant mortality by race and between 0.65 (North Dakota) to 0.00 (West Virginia) for unhealthy days by education. For some states, the choice of inequality aversion significantly impacts their PHPI value and state rank.

**Conclusions:**

Mean and inequality health outcomes can be combined into a single Population Health Performance Index for use by public and private policy makers, like the GDP is used as a summary metric to measure economic output. The index can allow for varying degrees of inequality aversion, an individual’s or jurisdiction’s value choice that can substantially impact the value of this new summary population health metric.

## Background

National and state health outcome goals are often framed in terms of improving the population mean and reducing or eliminating inequalities within the population. For example, in Healthy People 2020 [1] the two overarching goals are: 1) attain high-quality, longer lives free of preventable disease, disability, injury, and premature death, and 2) achieve health equity, eliminate inequalities, and improve the health of all groups. However as Keppel [2] pointed out with regard to Healthy People 2010, the first goal does not necessarily eliminate inequalities and improve the health of all groups. Different strategies are often needed for these two goals, and innovations often have higher impact at least initially in more educated or advantaged populations, which can at least temporarily increase such inequalities [3, 4].

A recent examination of what U.S. states have experienced in overall mean improvement in mortality rates compared to the improvement in the black-white mortality gap showed that between 1999 and 2013 there was no significant correlation between the mean measure and inequality measure reductions [5]. In this paper, it was observed that it is often the case states perform well on either the mean or inequality measure but struggle on the other.

If a policy maker was interested in trying to determine what would produce optimal results, some standard of what “optimal” means would need to be defined. As Wagstaff indicated with regard to his “achievement index”, such a summary metric would have to reflect a value judgment of the relative importance of mean improvement versus inequality reduction [6]. While Wagstaff’s achievement index is innovative, its complexity may inhibit some policymakers understanding of the metric. A simpler metric achieving the same goal of combining mean and inequality health outcomes into a single performance index could therefore add value. We therefore created a Population Health Performance Index (PHPI).

## Methods

Infant mortality data were extracted from the publicly available Center for Disease Control and Prevention’s Wonder’s Infant Death Database, accessible here: https://wonder.cdc.gov/. We collected the number of infant deaths, births, and infant mortality rates (deaths per 1000 live births) for all 50 U.S. states and Washington D.C. for blacks and whites of non-Hispanic origins, aggregated from the years 2011 to 2013. The following 12 states had fewer than 20 infant death events for blacks or whites over this time period and therefore were excluded from our analysis: Alaska, Washington D.C., Hawaii, Idaho, Maine, Montana, New Hampshire, New Mexico, North Dakota, South Dakota, Vermont, and Wyoming.

Unhealthy days data were extracted from the Center for Disease Control and Prevention’s Behavioral Risk Factor Surveillance System 2014 survey database. The unhealthy days measure is the self-reported number of days in the past 30 days that an individual felt physically or mentally unhealthy. We calculated the mean number of unhealthy days for all 50 U.S. states and Washington D.C. within four groups classified by educational attainment: less than high school, high school degree, some college, and a college degree.

For all states included in the analysis, the mean black infant mortality rate is 11.17 while that for whites is 5.17. The absolute inequality in rates ranges from 3.41 more infant mortality events per 1000 live births (Kentucky) or as high as 8.99 (Wisconsin). Mean infant mortality rates across all states range from 3.91 (Massachusetts) and 9.33 (Mississippi).

For unhealthy days by education, across all states the mean number of UHDs for college graduates is 3.68 and 6.05 for non-college graduates. The absolute inequality can be as low as 0.77 more unhealthy days per 30 days (North Dakota) or as high as 3.86 (West Virginia). Mean unhealthy days across all states range from 3.58 (North Dakota) or as high as 7.18 (West Virginia).

The population health performance index (PHPI) is a weighted average of two distinct indices: a mean index and an inequality index. Below we explain each index in detail. The mean index for state *i* is calculated as follows:

State Mean Index_i_ = 1- (Population Mean_i_ / Population Mean_Most Unhealthy State_).

The mean index takes a value between 0 and 1. A value of 1 represents a theoretically ideal outcome of no ill health events. For our two outcomes, a mean index value of 1 represents no infant mortality events or unhealthy days in the population. The mean index value for the state with the worst mean health outcome is 0. This bounds the mean index component between the ideal outcome value and the value for the worst state. For this analysis, we only consider blacks and whites, thus the mean outcome is not a national mean but rather a mean of the black and white populations aggregated.

Similarly, we calculated a state inequality index using the following formula for state *i*:

State Inequality Index_i_ = 1- (Inequality_i_ / Inequality_Most Unequal State_).

Again, the inequality index ranges from 0 to 1, with a value of 1 representing the ideal outcome of no inequality within a state and a value of 0 attributed to the most unequal state. We calculated both absolute and relative inequality and applied it to our methodology. Because we found no theoretical justification or substantial difference in the results, we present here the absolute inequality results and report the relative results on the PHPI website.

For infant mortality the state mean index has a mean of 0.32 with a maximum of 0.58 and the state absolute inequality index has a mean of 0.33 with a maximum of 0.62. There is no correlation between state mean and absolute inequality indices (correlation coefficient = 0.22). For UHD the state mean index has a mean of 0.27 with a maximum of 0.50 and the state absolute inequality index has a mean of 0.39 with a maximum of 0.80. There appears to be a relationship between a state’s mean index and absolute inequality index in the unhealthy days by education case (correlation coefficient = 0.76), but we do not investigate this finding further.

The state population health performance index (PHPI) was then calculated as a weighted average of the state mean index and state inequality index, with the weight representing the relative importance of the mean and inequality measures:

PHPI = (1-w) * State Mean Index + w * State Inequality Index.

where *w* takes a value between 0 and 1, indicating inequality aversion or mean- inequality trade-off.

A PHPI value of 1 is optimal as this value would signal no ill health events and no inequality within a population. The worst value of the PHPI is 0, which would indicate a state had both the least healthy mean and the greatest inequality.

If the user values mean population health outcomes equal to the health inequality within the chosen population, the mean index and inequality index would both be weighted by 0.5. For greater inequality aversion, the weight attached to the inequality index can be increased at the cost of decreasing the weight attached to the mean index. Likewise, for greater emphasis on the mean population health outcome, or less inequality aversion, the weight attached to the inequality index can be decreased in order to increase the weight attached to the mean index. In this analysis we assign weights of w = 0.9, 0.75, 0.5, 0.25, and 0.1 to signal strong, moderately strong, neutral, moderately weak, and weak inequality aversion respectively. We have developed an interactive website which contains all the underlying data for each state, and which allows the inequality aversion weight to be altered, showing the resulting score and rank changes in comparison to other states [7].

## Results

**Fig. 1:**
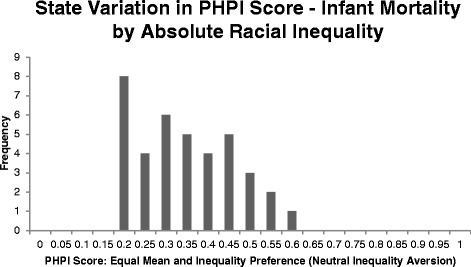
Using the Population Health Performance Index, most states have substantial room to improve on infant mortality by race

**Fig. 2:**
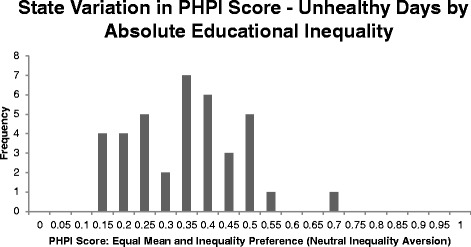
Using the Population Health Performance Index, most states have substantial room to improve on unhealthy days by educational inequality

The results for the PHPI score (neutral 50:50 weighting) are summarized in Figs. 1 and 2. For infant mortality by race, the mean PHPI score is 0.32, with a range from 0.60 (Massachusetts) to 0.17 (Delaware) when measuring infant mortality by race. For unhealthy days by education, the mean PHPI score is 0.33, with a range from 0.65 (North Dakota) to 0.00 (West Virginia).

Using the Population Health Performance Index, most states have substantial room to improve on infant mortality by race.

Using the Population Health Performance Index, most states have substantial room to improve on unhealthy days by educational inequality.

The variation across states in this figure shows that even with equal weighting of mean and inequality, all states have significant room for improvement (a PHPI of 1.00 indicates no negative health events or inequality in them, not simply the less challenging goal of having the best state performance). For infant mortality by race, even the best state, Massachusetts, has a PHPI equal to 0.60 despite having the lowest combined-race infant mortality rate (3.91 infant deaths per 1000 live births) and second lowest absolute inequality (3.46 more infant deaths per 1000 live births for blacks than whites). For unhealthy days by education, the highest PHPI with neutral inequality aversion is North Dakota (PHPI = 0.65), which signals it still has room to improve despite having the healthiest mean (3.58 unhealthy days in the past 30 days) and the lowest inequality (0.77 more unhealthy days in the past 30 days for non-college graduates than college graduates).

We also generated national maps of the quartile distribution of PHPI scores for different weights, which are available on our website.7 Some states with low means but low disparities, such as Mississippi, and states with high racial inequality but high means, such as Wisconsin, perform poorly when measuring both the mean and inequality using the PHPI.

As previously indicated, the weighting of mean and inequality components is a value choice; the 50:50 default weighting reflects equal weighting or no preference for either the mean or the inequality component. However jurisdictions or individuals may have a preference for one or the other. We therefore calculated the impact on PHPI scores of different weighting combinations: weak inequality aversion (w = 0.1); moderately weak aversion (w = 0.25); neutral aversion (w = 0.5); moderately strong aversion (w = 0.75); and strong inequality aversion (w = 0.9).

The direction and magnitude of the change in score between weights for a given state depends entirely on the difference between that state’s mean index and inequality index. The closer the mean index and inequality index are to each other for a given state, the less the weight on each component will matter in determining its PHPI. In other words, if a state performs equally well on both mean and inequality, the weight attached to each does not impact one’s valuation of that state’s population health performance. However, if a state performs much better on one dimension than the other, the weight attached to each can significantly impact one’s valuation of that state’s population health performance. Therefore, for those with strong inequality aversion, states with relatively better inequality indices than mean indices would appear to perform better as inequality is emphasized and states with relatively worse inequality indices than mean indices would appear to perform worse compared to an alternative valuation which does not place as strong as an emphasis on inequalities.

Using infant mortality by race as an example, an exploration of three states profiled in Table 1 illustrates the impact that inequality aversion can have on the user’s valuation of a state population health performance using the PHPI.

**Table 1:** PHPI State Examples – Infant Mortality by Absolute Racial Inequality

	Connecticut	Massachusetts	Mississippi
Black Infant Mortality Rate	10.21 (13th)	6.91 (1st)	12.40 (27th)
White Infant Mortality Rate	3.70 (3rd)	3.45 (2nd)	6.76 (37th)
Combined – Race Infant Mortality Rate	4.88 (7th)	3.91 (1st)	9.33 (39th)
Absolute Racial Inequality	6.51 (26th)	3.46 (2nd)	5.64 (15th)
State Mean Index	0.48 (7th)	0.58 (1st)	0.00 (39th)
State Inequality Index	0.28 (26th)	0.62 (2nd)	0.37 (15th)
PHPI – Weak Inequality Aversion	0.46 (8th)	0.58 (1st)	0.04 (39th)
PHPI – Neutral Inequality Aversion	0.38 (13th)	0.60 (1st)	0.19 (36th)
PHPI – Strong Inequality Aversion	0.30 (25th)	0.61 (1st)	0.34 (18th)

State examples of the Population Health Performance Index

Connecticut performs above average (State Mean Index = 0.48, 7th of 39 states) when considering the mean health outcome and below average (State Inequality Index = 0.28, 26th of 39 states) when considering health outcome inequality. When both are considered jointly and there is no preference for the mean versus inequality outcome, Connecticut would be judged as performing near the middle of the states (PHPI = 0.38, 13th of 39 states). When there is weak inequality aversion, Connecticut would be judged as performing well (PHPI = 0.46, 8th of 39 states), but a strong inequality aversion would reveal Connecticut as performing poorly (PHPI = 0.30, 25th of 39 states). The case of Connecticut shows how an individual or jurisdictional value judgment of mean versus inequality tradeoff, or inequality aversion, can greatly impact the valuation of Connecticut’s population health performance.

Massachusetts performs the best in the country (State Mean Index = 0.58, 1st of 39 states) when considering the mean health outcome and second best (State Inequality Index = 0.61, 2nd of 39 states) when considering health outcome inequality. When both are considered jointly and there is no preference for the mean versus inequality outcome, Massachusetts ranks first (PHPI = 0.60, 1st of 39 states). Similarly, when there is weak inequality aversion, Massachusetts would still rank first (PHPI = 0.58, 1st of 39 states); when there is strong aversion to inequality, it would again be assessed as performing the best in the country (PHPI = 0.61, 1st of 39 states).

Mississippi performs the worst in the country (State Mean Index = 0.00, 39th of 39 states) when considering the mean health outcome but performs moderately (State Inequality Index = 0.37, 15th of 39 states) when considering health outcome inequality. When both are considered jointly and there is no preference for the mean versus inequality outcome, one would rank Mississippi as performing poorly (PHPI = 0.19, 36th of 39 states). When there is weak inequality aversion, an individual would rank it at the bottom of the country (PHPI = 0.04, 39th of 39 states), but when there is strong aversion to inequality, an individual or jurisdiction would assess Mississippi as performing slightly above average (PHPI = 0.34, 18th of 39 states).

## Discussion

The Population Health Performance Index described here is the first attempt to create a measure combining mean and inequality components and apply it for U.S. states, building on the Wagstaff [6] concept that was applied to a sample of countries in 2002. Such an aggregate measure can be useful since the broad goals of population health policy are often stated to be improving overall health and reducing inequalities. The PHPI is designed to be easily understandable, having index scores range from 0 to 1, with 1 being the theoretically possible highest performance when combining the mean and inequality performance together and 0 being a state with both the least health mean and the highest inequality. Our initial analysis explored both mortality and non-mortality outcomes as well as racial and socioeconomic inequalities, but can be deployed using other health outcomes, stratifies, and populations. When mean and inequality are weighted equally, the PHPI shows infant mortality by race having a mean of 0.32 (range 0.17 to 0.60), and for equally weighted unhealthy days by education, the mean is 0.33 (range from 0.00 to 0.65). Since the best performing states only achieve scores of about 0.60, this indicates room for considerable progress in all states on this performance measure. Our analysis and website allows for different weights on the mean and inequality components of the PHPI since individuals and policy makers may value the two components differently. The direction and magnitude of the change in score between weights for a given state depends entirely on the difference between that state’s mean index and inequality index. If the state inequality index is significantly worse than the state mean index, like Connecticut, the state PHPI score will look worse for an individual or jurisdiction valuing inequalities more. If the state inequality index is considerably better than the state mean index, like Mississippi, a state will look better for a perspective which values inequalities more. If a state performs about the same on both measures, like Massachusetts, the state’s combined performance will not matter much with different perspectives on inequality aversion.

The strength of this study is its novel approach to combining mean and inequality outcomes into an easily understood, single metric like the Gross Domestic Product (GDP). In addition, we have applied it to two test cases involving different outcome and inequality domains. Weaknesses include having 12 states with insufficient African American deaths to allow for including in our analysis and low sample sizes in the unhealthy days measure, although these are of minimal concern at the state level of analysis.

This new metric, the PHPI, allows public and private policy makers to assess the performance of jurisdictions, like states, for mean and inequality outcomes together. We do not suggest that the summary measure should replace attention to the separate components, since the individual components may require separate policy approaches, and ignoring them could do disservice to either mean improvement or disparity reduction efforts. We hope and believe that such a summary index could become a recognized guide to performance and accountability such as the GDP has become for economic performance. The feature of being able to weight inequality aversion is critical, since such value judgments may vary across different population groups and will result in different values of the measure as we have demonstrated.

The PHPI does not address the important policy question of why states or nations have different results with regard to mean and inequality performance. It is of critical importance to determine which policies across all health determinants are most effective in moving each component, and in moving both together most efficiently [8]. Considerable cost effectiveness research will be needed to move this concept into practice, but it is an absolute underpinning of any realistic population health equity policy effort to move both mean and inequality outcomes and their PHPI performance together [9].

## Conclusions

The Population Health Performance Index (PHPI) is a new indicator of state population health combining both mean and disparity outcomes into a GDP like summary measure. We have demonstrated its application to both mortality and non-mortality outcomes as well as racial and socioeconomic inequalities, and in addition have shown how it varies with different degrees of inequality preference or aversion. We hope that such a summary index could become a useful recognized guide to population health performance and accountability along with other traditional measures.

## Abbreviations

GDP: Gross Domestic Product
PHPI: Population Health Performance Index
